# A novel mutation of thyroid hormone receptor β in exon 10 in a case of thyroid hormone-resistant non-Hodgkin’s lymphoma of the thyroid

**DOI:** 10.3892/ol.2014.2715

**Published:** 2014-11-20

**Authors:** KE CHEN, YANHONG XIE, LILING ZHAO, SHAOLI ZHAO, HONGHUI HE, ZHAOHUI MO

**Affiliations:** Department of Endocrinology, Third Xiangya Hospital of Central South University, Changsha, Hunan 410013, P.R. China

**Keywords:** thyroid resistance syndrome, thyroid non-Hodgkin’s lymphoma, thyroid hormone receptor β, mutation

## Abstract

Only a few previous studies have demonstrated an association between resistance to thyroid hormone (RTH) and thyroid cancer. The current study presents the case of a 67-year-old female who was referred to the Third Xiangya Hospital of Central South University with an enlargement of the neck that had grown gradually over two years and subsequently, rapidly enlarged over the two months prior to admission, alongside a slight sensation of shortness of breath. Laboratory data revealed a significantly increased level of thyroid-stimulating hormone (TSH), total triiodothyronine, total thyroxine, free triiodothyronine, free thyroxine, thyroprotein and thyroglobulin antibody; however, the levels of thyroperoxidase and TSH receptor antibody were within the normal ranges. A thyroid hormone suppression test revealed a TSH reduction of 32%, Magnetic resonance imaging of the pituitary gland was negative for abnormalities. The patient’s thyroid pathology revealed a non-Hodgkin’s lymphoma of the thyroid. CHOP + nimustine chemotherapy significantly reduced the clinical symptoms. The genetic analysis revealed a novel point mutation of the thyroid hormone receptor β (THRB) gene in exon 10 (g1680 G to A) in the 3′-untranslated region of the patient. To the best of our knowledge, this is the first case report of RTH with thyroid non-Hodgkin’s lymphoma, which involved a mutation (g1680 G to A) in exon 10 of THRB.

## Introduction

Resistance to thyroid hormone (RTH), also known as Refetoff syndrome, is a rare syndrome that manifests as reduced end-organ responsiveness to the thyroid hormone. The precise incidence of RTH is unclear. A study observed that high blood T4 levels were present in one case per 40,000 in neonatal screening (1). Patients with RTH exhibit elevated serum free thyroxine (FT4), free triiodothyronine (FT3) and normal or elevated serum thyroid stimulating hormone (TSH) levels. The characteristic clinical features vary, including an absence of the usual symptoms of hyperthyroidism/hypothyroidism, hyperthyroidism or hypothyroidism, with or without goiter (2). The majority of cases are related to thyroid hormone receptor β (THRB) mutations, a few cases are caused by thyroid hormone receptor α (THRA) mutations, and even fewer cases have no THR mutation, which may be associated with post transcriptional regulation (3–8).

Primary thyroid lymphoma (PTL) is a rare form of thyroid cancer, it accounts for 1–5% of all thyroid malignancies and 1–2% of all extra-nodal lymphomas. Typically patients present with a rapidly enlarging thyroid as opposed to other thyroid malignancies, about 30–50% of patients have complications with hoarseness, stridor, dysphagia and a pressure sensation in the neck (9).

Recent studies have reported that RTH is associated with certain types of thyroid cancer, including papillary thyroid carcinoma and papillary microcarcinoma (10–14). In the current study, we report a case of RTH with thyroid non-Hodgkin’s lymphoma.

## Case report

**Figure 1:**
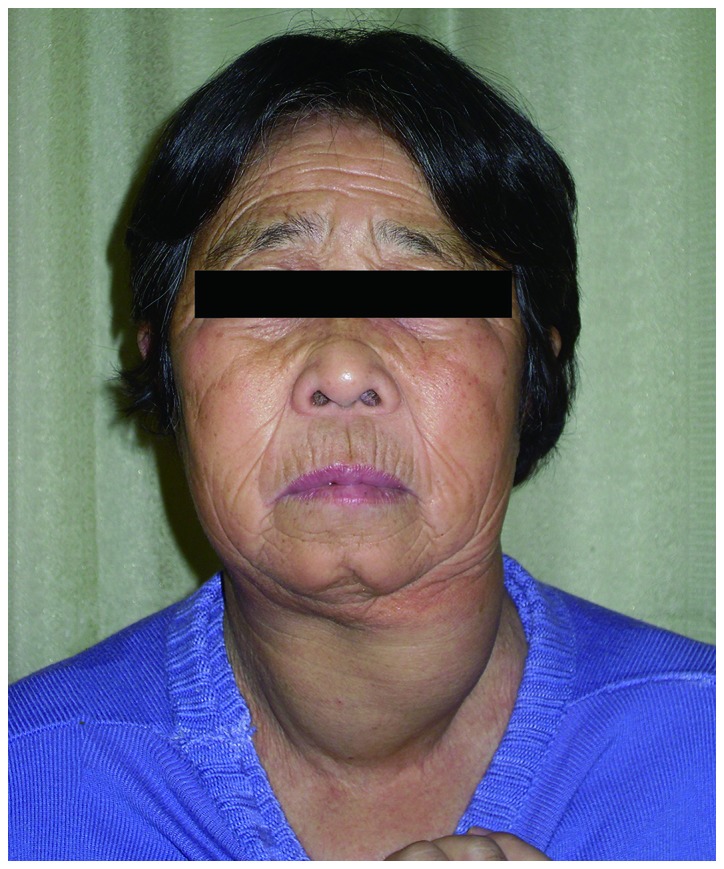
Physical examination revealed a third degree enlargement of the left lateral lobe of the thyroid. The right lateral lobe of the thyroid was normal.

**Figure 2:**
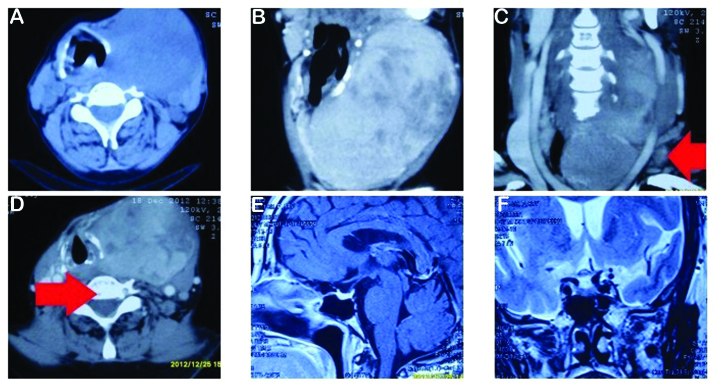
Neck CT and pituitary MRI images of the patient. Cervical CT revealed a heterogeneous mass of 7.3×5.3×9.2 cm in size in the left lateral lobe of the thyroid gland, a tracheal compression and shift to the right, lumen narrowing and multiple irregular low-density areas in mass (CT value about 44–100 HU). The right side of the thyroid was a normal size, with low density nodules. Neck CT images: (A) horizontal plane, (B) sagittal plane, (C) coronal plane (upper mediastinal lymph node enlargement, arrow head) and (D) cervical circular low density shadow (arrow head).Pituitary MRI of the patient did not reveal any pathological findings in either the (E) sagittal or (F) coronal planes. CT, computed tomography; MRI, magnetic resonance imaging.

Written informed consent was obtained from the patient and the patient’s family. A 67-year-old female was referred to the Third Xiangya Hospital of Central South University (Changsha, China) in December 2012 with a neck that had become gradually enlarged over the previous two years, with rapid enlargement in the previous two months, accompanied by a slight sensation of shortness of breath (Fig. 1). During the two years, no hyperthyroidism or hypothyroidism symptoms such as sensitivity to heat, irritability, tremors or sensitivity to the cold, fatigue and edema were experienced. No history of irradiation or family history of thyroid disease was reported. On admission, pulse rate was 82 bpm, regular blood pressure was 145/59 mmHg and body temperature was 36.6°C. Physical examination revealed a third degree enlargement of the left lateral lobe of the thyroid; the right lateral lobe thyroid was normal and no proptosis was present. Laboratory investigations revealed significantly elevated levels of serum TSH [33.63 μIU/ml; normal range (N), 0.27–4.2], total T3 (TT3; 3.11 nmom/l; N, 1.3–3.1), total T4 (TT4; >320 nmom/l; N, 66–181), thyroperoxidase (TPO) antibody (23.8 IU/ml; N, 0–34), TSH receptor antibody (TRAB; <0.3 IU/l; N, 0–1.75). In the outpatient clinic the following day, the thyroid hormone examination was repeated, yielding serum TSH values of 32.28 μIU/ml, TT3 of 3.58 nmom/l, TT4 of more than 320 nmom/l, free T3 (FT3) of 9.57 pmom/l (N, 3.1–6.8 pmom/l), free T4 (FT4) of >100 pmom/l (N, 12–22 pmom/l), TPO antibody of 13.56 IU/ml, TRAB of 1.3 IU/l, thyroglobulin antibody (TgAB) of 141.2 IU/ml (N, 0–115), thyroprotein (TG) of 192.3 ng/ml (N, 1.4–7.8). Blood cell count showed a white blood cell level of 13.8×10^9^/l (N, 4.0–10.0), hemoglobin level of 92 g/l (N, 110–150), platelet level of 233×10^9^/l (N, 100–300) and serum albumin of 34.2 g/l (N, 35.0–50.0). The thyroid color Doppler ultrasound scan revealed a hypoechoic mass on the left lateral lobe of the thyroid, while the right lateral lobe of the thyroid had an uneven echo. The additional color Doppler ultrasound results of the liver, gallbladder, pancreas, spleen, retroperitoneal lymph node, uterus and ovary, kidney, ureter, bladder and bilateral adrenal were all normal. A chest X-ray revealed a widened mediastinum, tracheal compression and cardiac enlargement (primarily an enlarged left ventricle). A cervical computed tomography (CT) showed a thyroid left lateral lobe tumor, considered to be thyroid cancer and possible bilateral neck metastases, multiple mediastinal lymph node metastases and cervical vertebra centrum bone shifts (Fig. 2A–D). Single photon emission computed tomography (SPECT) of the thyroid showed that the volume of the left lateral lobe had increased significantly and there were multiple bilateral thyroid nodules. Therefore, the possibility of a malignant tumor was considered. Bone marrow cytology examination showed obviously active bone marrow hyperplasia and normal cells in different stages. Magnetic resonance imaging (MRI) of the pituitary did not reveal any pathological findings (Fig. 2E–F).

A dexamethasone suppression test (2 mg q6h for two days) showed that the level of TSH had decreased from 35.53 to 16.3 μIU/ml, a thyroid hormone suppression test (thyroid tablets 100 mg qd for two days) revealed that the TSH had decreased from 32.58 μIU/ml to 18.26 μIU/ml. It was not possible to measure L-T3.

**Figure 3:**
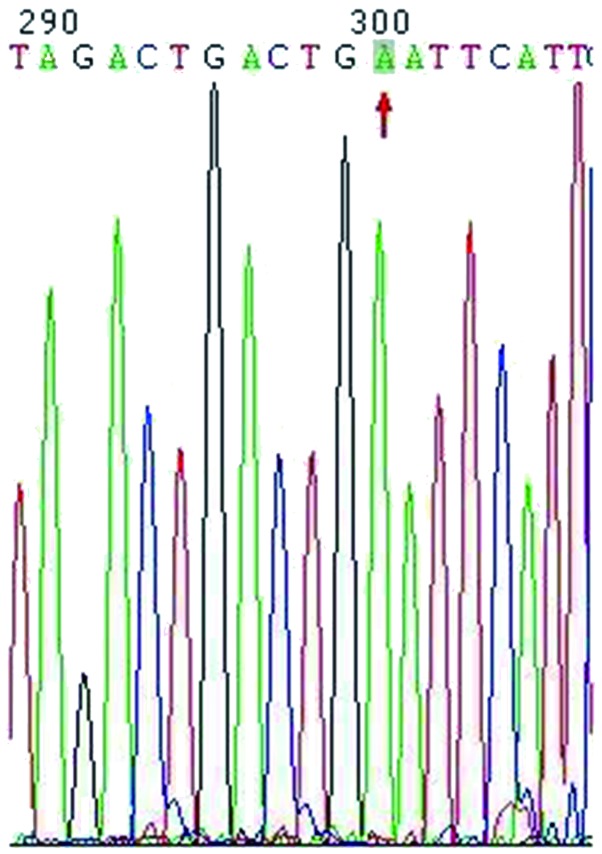
Sequence analysis of the thyroid hormone receptor β gene showing a 3′-UTR mutation (g1680 G to A) at exon 10 (red arrow). The same mutation was observed in the patient’s son and daughter.

**Figure 4:**
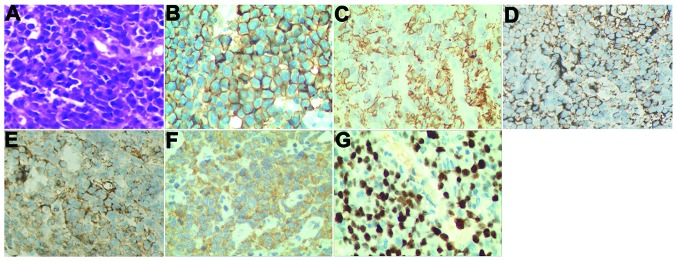
Tissue pathology staining was positive for B-cell and plasma cell surface markers. (A) Hematoxylin and eosin staining, and positive staining for (B) CD38, (C) CD20, (D) Kappa chain, (E) lambda chain, (F) Bcl-2 and (G) Ki 67.

The genetic analysis presented in Fig. 3 revealed that exons 1–9 were normal, and that exon 10 had a novel point mutation of THRB (g1680 G to A); the same mutation was identified in the patient’s son and daughter. To exclude the mutation as a THRB polymorphism, the sequences of 100 healthy controls were analyzed and the mutation was not found (data not shown). A core biopsy, stained with hematoxylin and eosin (HE), showed round and polygon poorly differentiated tumor cells (Fig. 4A). The results of immunohistochemical staining were as follows: CD20 (+), CD38 (+), immunoglobulin light chain [Kappa (+) and Lambda (+)], BCL-2 (+), CD79α (−), CD34 (−), CD3 (−), CD5 (−), CD10 (−), CKPan (−), EMA (−), CD30 (−) and TdT (−) (Fig. 4). Ki67 stained ~40% of the cells. These results indicated a non-Hodgkin’s lymphoma [lymphoplasmacytic lymphoma (LPL)]. The patient’s serum showed an IgA level of 1.05 g/l (N, 0.5–5 g/l), an IgE level of 31 IU/ml (N, 0–385 IU/ml), an IgG level of 7.8 g/l (N, 5.16–14.25 g/l) and an IgM level of 1.13 g/l (N, 0.3–2.09 g/l); no M protein was identified by protein electrophoresis.

The patient was transferred to the department of hematology for CHOP + nimustine chemotherapy, and, following a course of treatment, the neck swelling was significantly reduced and shortness of breath was improved. The patient and son refused the subsequent treatment and the collection of blood samples from other family members, therefore no clinical and genetic data could be obtained for the family.

## Discussion

Due to a low morbidity and complicated clinical manifestations, RTH is often misdiagnosed. Prior to diagnosis of RTH, alternative causes of elevated TT4 and TSH must be excluded, for example, raised serum binding proteins, non-thyroidal illness (including acute psychiatric disorders), drug use (amiodaron, heparin), familial dysalbuminemic hyperthyroxinemia and TSH secreting pituitary adenoma (TSH-oma); the most difficult distinction is between RTH and TSH-oma (15). In the current case, non-thyroidal illness, increased albumin and drugs were excluded. The patient’s TT4, TG and TGA levels were significantly increased, however, the levels of FT3 and FT4 were also increased, which aided in excluding elevated TT4 due to increased serum TGB. A thyroid suppression test showed that TSH was reduced by 32%, pituitary MRI was negative and further the genetic analysis supported the view that the patient had RTH.

RTH is a rare autosomal, hereditary disease, consisting of 75–85% familial cases and 15–25% sporadic cases, with THRB mutations causing RTH in ~90% of cases (16). In the current study, the patient, and the patient’s son and daughter all possessed a novel point mutation in exon 10 (g1680 G to A) of THRB. The point mutation was located in the 3′-untranslated region (UTR) rather than in the coding sequence (CDS), however, this mutation may affect the post-transcription processing of THRB.

Patient pathology revealed that this case did not belong to the most common type of PTL, rather, it was indicated to be a LPL (17). LPL is an extremely rare subtype of non-Hodgkin’s lymphoma, which is a low-grade, B-cell neoplasm composed of small lymphocytes, plasmacytoid lymphocytes and plasma cells that typically involve the bone marrow, lymph node or spleen. However, a few cases do not originate in the bone marrow but are extramedullary. If the extramedullary tumor infiltrates bone marrow, it may cause pancytopenia, organomegaly and hyperviscosity; the majority of cases are asymptomatic or present with anemia. The pathological features usually present as eccentric nuclei and a more abundant basophilic cytoplasm. The typical immunophenotype of LPL shows expression of CD19, CD20, CD22, FMC7, BCL2, CD38 and CD79a; however, CD5, CD10, and CD23 are usually absent (18). In the current case a primary tumor was not derived from bone marrow and non-Hodgkin’s lymphoma did not infiltrate the bone marrow; although the CT scan indicated possible cervical bone metastases, only mild anemia was detected, the bone marrow cytology did not suggest lymphocyte and plasma cell infiltration. Additionally, M protein was not detected and the Ig M level was within the normal range. The immunohistochemical staining supported the diagnosis of thyroid LPL (10–14).

A number of studies of RTH with thyroid cancer have been previously reported (10–14). In an animal study, mutation of THRB significantly increased the morbidity of spontaneous thyroid carcinoma through the activation of TSH-mediated signaling pathways (19). Whether or not the mutation of THRB increases the morbidity of non-Hodgkin’s lymphoma or LPL remains unclear.

In conclusion, to the best of our knowledge this is the first report of a case of RTH with thyroid non-Hodgkin’s lymphoma. The pathology may be considered as LPL, which is an extremely rare subtype of non-Hodgkin’s lymphoma. The present case indicated that the point mutantion of THRB in 3′-UTR might be an important indicator for RTH or non-Hodgkin’s lymphoma. Further functional studies will be performed in order to confirm the function of this THRB mutation in RTH or non-Hodgkin’s lymphoma.
